# Twenty years of genome-wide association studies: Health translation challenges and AI opportunities

**DOI:** 10.1038/s41431-025-01951-5

**Published:** 2025-10-14

**Authors:** Jie Huang, Gary R. McLean, Andre Franke

**Affiliations:** 1 School of Public Health and Emergency Management, Southern University of Science and Technology, Shenzhen, China; 2 Institute for Global Health and Development, Peking University, Beijing, China; 3 National Heart and Lung Institute, Imperial College London, London, UK; 4 Institute of Clinical Molecular Biology, University of Kiel, Kiel, Germany

**Keywords:** Genetics, Diseases

## Abstract

A landmark genome-wide association study (GWAS) in 2005 led to a major discovery about the genetics of age-related macular degeneration. Since then, thousands of GWAS have been published and tens of thousands of genomic loci have been reported for association with human traits ranging from established ones (e.g. height, cardiovascular disease) to unconventional ones (e.g. same-sex sexual behavior, family income). While some claim that GWAS has already fulfilled its promises, we argue that it has yet to fully showcase its power in unraveling the secrets of the human genome and its links to phenotypes. The March 2025 bankruptcy of 23andMe serves as a stark reminder of the limited translational value of GWAS to the general public. The GWAS research community can achieve more only if we begin with a sober and objective assessment. Here, we first outline “*Four Persistent Obstacles”* that continue to hinder GWAS progress and discuss how a “*Global Research Ecosystem”* may be well-positioned to overcome them. We also highlight the transformative rise of artificial intelligence (AI) exemplified by AlphaFold’s unprecedented power in predicting protein structures. Finally, we introduce a novel concept, the “*trait efficiency locus* (TEL)”, as a complement to the widely used quantitative trait locus (QTL) framework, providing a new lens for evaluating genetic discoveries. One could also term it “structural trait locus (STL)”, but “TEL” emphasizes the central idea that efficiency is what ultimately matters.

## Twenty years of GWAS

On April 15, 2025, Southam et al. published a brief In Retrospect piece titled *“Twenty years of genome-wide association studies”* [1]. It began by citing the landmark study by Klein et al. [2] published in 2005, thus marking 2025 as the 20th anniversary of genome-wide association studies (GWAS). Five years earlier, Loos et al. commemorated the 15-year milestone with a review [3], also counting the Klein et al. study as the starting point of the GWAS era.

Both Southam et al. and Loos et al. acknowledged that the Wellcome Trust Case Control Consortium (WTCCC) study published in 2007 was the real catalyst for the explosion of GWAS in the field [4]. Consequently, an international group led by Prof. Peter Visscher considered 2007 as the starting point of the GWAS era instead. This group published the “*Five Years of GWAS Discovery*” [5] in 2012, followed by “*10 Years of GWAS Discovery: Biology, Function, and Translation*” [6] in 2017 and “*15 years of GWAS discovery: Realizing the promise*” [7] in 2023. The slight delay in the latest review (2023 instead of 2022) may be attributed to COVID-19 disruptions, or perhaps because statisticians, unlike mathematicians, are less obsessed with precise numbers and strict anniversaries. Regardless, we anticipate that a “*20 years of GWAS discovery*” review from this group will likely appear around 2027.

The 2025 In Retrospect piece by Southam et al. was relatively brief (under two pages), and about half of the text and the sole figure focused on recounting the Klein et al. study [2]. This underscores the need for a more in-depth reflection on two decades of GWAS, particularly one that evaluates its real-world health impact and explores the transformative potential of emerging artificial intelligence (AI).

The 15-year review by Loos et al. stated that approximately 4500 GWAS had been published, covering nearly 5000 traits and diseases, prompting the optimistic title *“No signs of slowing down.”* Since GWAS has clearly established that most complex traits are highly polygenic or even omnigenic, this necessitates larger sample sizes, advanced analytical methods, and therefore no reason for slowing down. However, the past five years have seen a noticeable decline in traditional GWAS publications. This trend reflects both a shift toward sharing GWAS results without formal publication and a rising bar for novelty and rigor when publishing GWAS.

## From discovery to impact: a reality check on health translation

We fully recognize the large volume of discoveries delivered by GWAS. For example, GWAS has: (i) linked *IL6R* variants to C-reactive protein (CRP) levels and motivated trials of tocilizumab and other IL6R antagonists in rheumatoid arthritis and coronary disease [8]; (ii) highlighted *CYP2C19* polymorphisms affecting clopidogrel metabolism and led to genotype-guided antiplatelet therapy recommendations [9]; (iii) identified lipoprotein(a) variants that informed the development of antisense therapies targeting apolipoprotein(a) [10]. However, these examples still fall short of the levels reached by other blockbuster drug developments such as GLP-1-based therapy for obesity that was recognized by the 2024 Lasker Award [11]. The PCSK9 target that revolutionized lipid-lowering therapies was discovered in 2003, before the GWAS era began if we consider 2005 as the birth year of GWAS [12, 13]. As a reality check, we should ask: how many drug discoveries can be directly attributed to GWAS? A 2023 review identified 40 germline genetic findings that led to novel approved drugs, with 36 for rare conditions and only 4 for common diseases. Strikingly, even not all of these 4 findings for common diseases were initiated by GWAS [14].

Consider the 2022 study that identified 12,111 independent SNPs for height, capturing nearly all of the common SNP-based heritability [15]. Statistically, it is an extraordinary feat, validating the infinitesimal model first proposed by Ronald Fisher over a century ago [16]. Yet, from a translational perspective, the question remains: what practical benefits or applications does this offer? Could it help parents of average height but who are concerned about their children’s short stature? Similarly, while the findings from GWAS on body mass index (BMI) abound [17], how many of them can directly inform clinical decisions such as whether to take a blockbuster weight-loss medication? [18]

Polygenic risk scores (PRS), one of the most touted tools that were born out and raised by GWAS, have been reported to predict individual risk for numerous diseases [19]. In some cases, such as for coronary artery disease, the predictive power of PRS exceeds that of traditional risk factors [20]. Given that DNA can be obtained at birth or even pre-implantation [21], this opens possibilities for early intervention. Yet, the March 2025 bankruptcy of 23andMe, once the flagship of direct-to-consumer (DTC) genomics, serves as a stark reminder of the limited translational value of GWAS and public appetite for PRS-based testing [22]. If such a once-in-a-lifetime genome test costs no more than a once-in-a-year routine physical exam, why aren’t more people buying it and taking it seriously?

## Four persistent obstacles

Despite tremendous progress, several foundational challenges persist in GWAS over the past two decades:

### 1). Technological inertia

One big legacy from the Human Genome Project (HGP) is the Genome Reference Consortium (GRC) for the human genome (GRCh). Although GRCh38 assembly was released in 2013, GRCh37 (released in 2009) remains the default reference genome for most GWAS summary statistics. The latest telomere-to-telomere (T2T) and pangenome assemblies are expected to offer richer genomic resolution, but their delayed adoption at scale would be expected. Although a 2023 paper rightly stated in its title that “*every base everywhere all at once: pangenomics comes of age*” [23], widely used tools like PLINK [24] and PheWeb [25] still rely on the restrictive REF/ALT format, restricting accurate representation of structural variants and pan-genomic diversity. The time when pangenomics is widely used might still be ages away.

### 2). LD bottleneck

Linkage disequilibrium (LD) continues to hamper post-GWAS analyses. Using the style of the pangenome publication title mentioned above, we could bluntly frame this situation as “*every locus everywhere all at once: LD out of control*”. There is a lack of synergized solutions to address LD [26]. For example, popular software like LDSC [27], LDPred [28], LDGM [29] all come with their own LD reference files and formats, and there is a lack of portability and scalability. As sequencing resolution improves and more diverse populations are studied, compulsory reliance on massive LD matrices is becoming computationally burdensome. LD has been an engine for genotype imputation which in turn has become a key force in driving GWAS to a cost-effective scale [30], but we must now revisit the bright and dark sides of LD, also the reason for its popularity over the past and the alternatives for its future. In the future, shall we move towards a 3-billion-by-3-billion LD matrix for every population, or adopt a deep learning model that could learn LD patterns and generate relevant matrices like ChatGPT without explicit enumeration?

### 3). Prioritizing heritability over actionability

The obsession with closing the “missing heritability” gap has distracted GWAS researchers from improving the clinical utility. Let’s return to the height GWAS example with over 12,000 SNPs discovered. It may explain well the phenotypic variance at the population level. But we now need to also explain well at the individual level, which includes individual SNP and individual subject. This is akin to a statistical analysis that shows all ~19,000 mayors and their close circles across  all municipal governments in the USA (including cities, towns, boroughs, and villages) could explain half of the public opinion of ~340 million US citizens. While statistically fascinating, the scientific or practical value for such information would be limited for a presidential candidate who has limited time and resources to make actionable strategies to win the race. The goal must shift from heritability to actionability.

### 4). Inadequate samples for diversity, equity, and inclusion (DEI)

Although it is debatable to keep a certain percentage of racial profile in government or enterprise workforce, it is unquestionably necessary, from a scientific standpoint, to include people with diverse evolutionary backgrounds in GWAS. A 2016 paper titled *“Genetic Misdiagnoses and the Potential for Health Disparities”* highlighted how under-representation of diverse ancestries can lead to false pathogenic classifications [31]. Over 80% of GWAS participants have European ancestry, creating major limitations for generalizability and equity. The expansion of GWAS into Africa, South America, and Asia is both a scientific and moral imperative. Diverse cohorts will not only enable more inclusive polygenic prediction but will also uncover population-specific biology and gene-environment interactions.

## New journey: a global research ecosystem for better GWAS

Despite persistent challenges mentioned above, the international research community is ready for better GWAS research that could potentially bear more fruit for health translation. Unexpectedly, the COVID-19 pandemic accelerated this momentum.

### 1). Many people feel involved

The unprecedented scale of sample collection for SARS-CoV-2 testing during the COVID-19 pandemic made the public far more familiar with genetics and genomic testing. Also, GWAS has spanned a wide spectrum of traits—from diseases like type 2 diabetes and Alzheimer’s to controversial topics such as same-sex sexual behavior [32] and family income [33]. Although the biological interpretation and practical implications of some of these findings remain unclear, they have sparked public interest and demonstrate the inclusive, far-reaching nature of GWAS research. Beyond health, GWAS has profoundly contributed to our understanding of human population history and adaptation. Fine-scale ancestry maps and signals of natural selection have been inferred using GWAS data [34]. For instance, studies have explored polygenic adaptation of height over the past 2000–3000 years [35], and the genetics of skin pigmentation in East Asian populations [36].

### 2). Many disciplines could contribute

Another positive side of the COVID-19 pandemic is that it catalyzed broader engagement with bioinformatics and genetics across diverse research areas. Beyond its discoveries, GWAS has driven major methodological innovations. The need for scalable, user-friendly tools has drawn in researchers from diverse disciplines including statistical genetics, computer science, and epidemiology. Mendelian Randomization (MR) [37] has flourished in the GWAS era, enabling the use of genetic variants to infer causal relationships between exposures and disease outcomes. MR has informed public health guidelines and helped prioritize drug targets, often even before clinical trial data becomes available.

### 3). More global biobank engines to come

The UK Biobank (UKB) [38] and China Kadoorie Biobank (CKB) [39]—two landmark population studies from the West and East—were both launched around 2003, shortly after the SARS outbreak. In the aftermath of the COVID-19 pandemic, a new generation of biobanks is rapidly emerging. One silver lining of the pandemic was the global scale-up of genetic testing, which showed the public the value of conducting biological sampling at scale and with ease. This unprecedented mobilization has increased public and political recognition of the value of large-scale biobanking. Going forward, national and regional biobank initiatives, especially in underrepresented populations, are likely to accelerate, laying a more globally representative foundation for genetic research.

### 4). More innovative technologies to emerge

The COVID-19 crisis also catalyzed rapid innovation in sequencing technologies and data sharing frameworks. The PANGO [40] lineage system and GISAID [41] demonstrated how open, collaborative infrastructure can power real-time tracking and analysis of viral evolution. These systems, built during a crisis, have set a precedent for affordable, scalable genomics. As sequencing costs continue to decline and cloud-based bioinformatics becomes more accessible, we anticipate that cutting-edge genomics will no longer be limited to well-funded institutions. Instead, it will empower researchers and clinicians worldwide, further democratizing the benefits of GWAS and precision medicine.

## The AI revolution: a catalyst for genomic translation

While GWAS began as a statistical approach, AI is now redefining its future. With the explosion of multi-modal data including genomic, imaging, wearable, and clinical data, AI excels in extracting patterns across dimensions and scales that classical methods cannot handle. AI models, especially large language models, are increasingly applied to genomics. After all, the human genome is the largest known language, consisting of billions of letters.

The expression “*C-change*” is often considered a misheard version of the correct idiom “*Sea change*”. In that spirit, AlphaFold’s unprecedented ability to predict the three-dimensional structure of proteins represents a true “*S-change*”, that is, a structural revolution in biology. Structure, not just quantity, holds the key to understanding function, much like how an organization’s hierarchy influences its performance more than its headcount. Whether for drug design or understanding the transmissibility of the SARS-CoV-2 Omicron variant, the structure (rather than the sequence) is crucial to understanding biology [42]. The 2024 Nobel Prizes in Chemistry reinforced this shift, by awarding breakthroughs in protein structure prediction, transitioning from sequence to structure-based biology [43]. The Nobel in Physics honored foundational work in neural networks, providing a theoretical backbone for today’s deep learning revolution [44].

Besides the high-profile success of DeepMind and its flagship AlphaFold, an army of “Deep” and “Alpha” is transforming life sciences at unprecedented speed. To name a few, this includes AlphaMissense [45], DeepVariant [46], DeepSweep [47], DeepMR [48]. Many biobanks now include imaging, electronic health records, and free-text clinical notes. Natural language processing (NLP) and computer vision techniques allow extraction of structured phenotypes at scale. AI models are being used to derive endophenotypes from retinal scans and wearable devices, which can then serve as high-resolution phenotypes for GWAS.

Finally, AI is especially powerful in integrative analysis. Multi-omics data (e.g., gene expression, epigenetics, proteomics, metabolomics) can be combined with genotype and phenotype data to build predictive models. Graph neural networks, variational autoencoders, and multi-modal transformers are being developed to connect variants to genes, pathways, tissues, and eventually, diseases. These models offer a systems-level understanding of how genetic variation propagates.

## “Efficiency”: A revisit and reflection

Since early 2025, after the establishment of the U.S. Department of Government Efficiency (DOGE), “*Efficiency*” has become a political buzzword [49]. Research funding cuts in the name of “government efficiency” pose serious challenges to researchers worldwide. It is not our intention, nor within the scope of this writing to discuss pros and cons of pursuing government efficiency. However, we do point out that the functioning of human society shares many similarities with nature. Terms like ‘*regulation*’ and ‘*expression*’ used in genomics are also commonly used in government documents with comparable meanings. Whether it is government efficiency or biological efficacy, three-dimensional structure usually matters more than one-dimensional numbers (*i.e*., quantities).

In the real world, where countless individuals interact, the input-output relationship is far from linear. An ancient Chinese parable illustrates this well: adding more monks does not necessarily increase the amount of drinking water available in a temple. The story goes: *one monk carries two buckets of water* (one bucket on each side of a wooden stick); *two monks carry one bucket of water* (one monk at each end of a wooden stick); *three monks carry no water* (each thinking the other two would or should do it). This parable becomes more interesting and complex as more monks are added. Nevertheless, the key takeaway is that higher efficiency requires a deeper understanding of the complex inter-individual interactions that drive outcomes.

Apparently, the parable above could not be simply used to justify “less is more”. As the philosopher Georg Wilhelm Friedrich Hegel once wrote, “*What is real is rational*.” Maybe three monks don’t get more drinking water than one single monk, but the other two monks certainly would have value for something else. Take the human body and human genome, for example. The human appendix was once considered a vestigial organ with no clear function. Similarly, over 90% of the 3 billion DNA nucleotides in the human genome were once labeled “junk DNA” because they do not code for proteins. However, later research revealed that non-coding DNA plays crucial roles in regulating gene expression and maintaining genomic stability. This insight encourages us to rethink what might initially seem “inefficient” or “useless.” We hope that efforts by DOGE and similar organizations recognize the long-term value of overlooked components, just as biology has revealed the essential roles of previously ignored DNA regions.

Traditionally, medical and biological researchers have studied genomics using metrics like Quantitative Trait Locus (**QTL**) [50]. It refers to genetic loci associated with quantitative traits, such as body mass index, blood cell counts, or gene expression levels. A search for “*quantitative trait locus*” on Google Scholar returns over 1.6 million records. However, numerical measurements are not always sufficient or accurate. Many quantitative traits, like BMI or blood counts, don’t always follow a straightforward linear dose-response relationship with health outcomes. Quantity alone is not the most important factor; instead, it is “efficiency” that determines whether a trait is beneficial or detrimental.

**Fig. 1:**
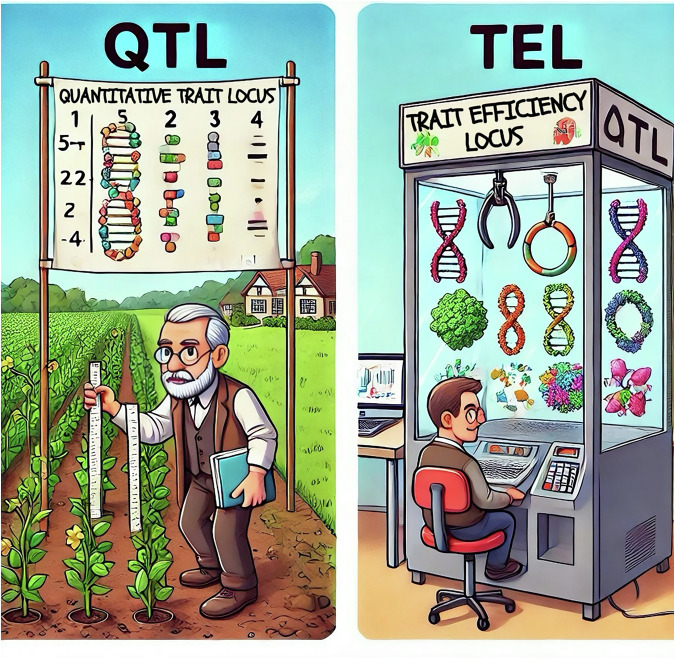
Illustration of the difference between QTL and TEL. This AI-generated figure illustrates “AI opportunities” by contrasting QTL and TEL in a cartoon style. On the left, an old-fashioned gentleman uses rulers to measure “quantity”; on the right, a computer geek operates a claw machine. The claw-machine metaphor represents molecular binding and interaction, which are largely determined by 3D structure and lead to variation in “efficiency”.

We therefore propose a new term, “trait efficiency locus (**TEL**)”, as a supplementary metric for QTL. A “TEL” is driven by interacting molecular structures that determine how efficiently a trait is realized. One could term this a structural trait locus (**STL**), but TEL emphasizes the central idea that efficiency is the outcome that ultimately matters. Take the high-sensitivity CRP protein for example. Since the beginning of this century, a Boston research group has been reporting CRP as one of the strongest predictors of future coronary events [51, 52], yet the lack of evidence for a causative role has been repeatedly reported [53]. As a matter of fact, no clinical trial to date has directly targeted CRP itself. We hypothesize that this perplexity arose because only “quantity,” not “efficiency,” has been targeted. Classical biology states clearly that missense mutations change protein composition and structure, which in turn affects efficiency. Yet, current protein measurement technologies still largely focus on quantity (pQTL) rather than structural or functional efficiency. The structural differences among subtypes of most proteins are generally subtler than those among the proteins for the ABO blood group. But on the other hand, we could imagine that every protein has its own subtle structural differences that might lead to completely different effects. Previously, we drew a claw toy grabber machine to illustrate the grabbing power of human ACE2 receptor by the SARS-CoV-2 spike protein [54]. We now utilized AI to draw a comparison between the traditional QTL and our newly proposed concept of TEL (Fig. 1). “Efficiency” here is represented by the grabbing success rate, determined more by the structure than the quantity of either the machine claws or the toys.

## Concluding remarks

After 20 years, GWAS has matured into a foundational tool for population genomics and human genetics. It has revealed the polygenic nature of most traits, enabled biological insights, and opened doors for clinical translation. But challenges remain: technical, interpretive, and ethical. AI brings the most exciting opportunities to overcome these hurdles. AI can scale with data, uncover hidden patterns, and facilitate integration across diverse modalities. The distinguished scientist Francis Collins once published two books: *The Language of Life* and *The Language of God*. With the excitement around large language models, it’s worth recalling that no “language” looms larger than the genome with billions of letters. The integration of GWAS, AI, and real-world data has the potential to redefine health care. It ranges from predicting disease risk and guiding preventive strategies, to designing personalized therapies and empowering individuals to manage their own health. The next decade will determine how close we come to realizing this vision.
